# SOCIAL PARTICIPATION OF TRANSGENDER BABY BOOMERS IN QUÉBEC: RESULTS FROM A CASE STUDY OF ACTIVISTS

**DOI:** 10.1093/geroni/igae098.0099

**Published:** 2024-12-31

**Authors:** Dolores Majón Valpuesta

**Affiliations:** Université de Sherbrooke, Sherbrooke, Quebec, Canada

## Abstract

The systematic discrimination linked to the intersection of gender identity and advanced age in transgender older adults results in further social participation restrictions and higher risk of disability (65% of transgender adults report poor mental health). Despite the importance of participation for health, little is known about the social participation of transgender older adults, especially among the new generation, the baby boomers. This study thus aimed to explore the social participation for transgender older adults. Based on the Iridescent Life Course theory, a case study was carried out with 2 transgender baby boomer community-dwelling from Quebec (Canada). Two French-speaking transgender women, 60 and 67 years old, with more than 5 years of activism. According to the experience of these activists, participation was positive and, with the gender transition in old age, a form of liberation, overcoming fears and denunciation, having a favourable impact on the community (e.g., sharing life experience for empowering other generations). Despite their positive experience, gender transition also involved a networks restriction, and, in some situations, they considered their rights were not respect. Presenting themselves with pride allows them to overcome the stigmatization of others as a barrier to participate. Boomers are perceived as a prejudiced generation and, consequently, activists were more comfortable interacting with younger generations (more naturalization, less judgmental). When trying to be included in heteronormative and cisgender contexts that did not consider the specificity of their life trajectories as they age, transgender older adults perceived the invisibility of their realities conditioned their social participation.

